# Signaling Pathways That Regulate Normal and Aberrant Red Blood Cell Development

**DOI:** 10.3390/genes12101646

**Published:** 2021-10-19

**Authors:** Mark C. Wilkes, Aya Shibuya, Kathleen M. Sakamoto

**Affiliations:** Division of Hematology/Oncology, Department of Pediatrics, Lucile Packard Children’s Hospital at Stanford University, Palo Alto, CA 94305, USA; mwilkes@stanford.edu (M.C.W.); ashibuya@stanford.edu (A.S.)

**Keywords:** kinases, cell signaling, erythropoiesis

## Abstract

Blood cell development is regulated through intrinsic gene regulation and local factors including the microenvironment and cytokines. The differentiation of hematopoietic stem and progenitor cells (HSPCs) into mature erythrocytes is dependent on these cytokines binding to and stimulating their cognate receptors and the signaling cascades they initiate. Many of these pathways include kinases that can diversify signals by phosphorylating multiple substrates and amplify signals by phosphorylating multiple copies of each substrate. Indeed, synthesis of many of these cytokines is regulated by a number of signaling pathways including phosphoinositide 3-kinase (PI3K)-, extracellular signal related kinases (ERK)-, and p38 kinase-dependent pathways. Therefore, kinases act both upstream and downstream of the erythropoiesis-regulating cytokines. While many of the cytokines are well characterized, the nuanced members of the network of kinases responsible for appropriate induction of, and response to, these cytokines remains poorly defined. Here, we will examine the kinase signaling cascades required for erythropoiesis and emphasize the importance, complexity, enormous amount remaining to be characterized, and therapeutic potential that will accompany our comprehensive understanding of the erythroid kinome in both healthy and diseased states.

## 1. Introduction

Aberrant kinase activation contributes to the pathogenesis of many diseases, including hematopoietic disorders, and pharmacologic inhibition of these kinases has become a major therapeutic strategy in the management of these diseases [1,2,3,4,5,6]. Indeed, the first protein-targeting therapy (imatinib mesylate, sti-571, or Geevec^®^) was designed to inhibit the kinase activity of the fusion gene *Bcr-Abl* in chronic Myeloid Leukemia [7]. As of 31 March 2021, there are 65 small molecule kinase inhibitors approved by the Food and Drug Administration (Drugs@FDA). 

Diamond Blackfan Anemia (DBA) results from genetic mutations in one of at least 20 different ribosomal genes [8]. These mutations are carried by every cell of the patient, and it is poorly understood why these mutations severely impact erythropoiesis with such specificity [9]. Pure red blood cell aplasia is due to a restriction of the earliest committed erythroid progenitors, manifesting as reduced erythrocytes [10]. Ribosomal stress due to ribosomal insufficiency, increased p53 activity [11,12] and reduced *GATA1* transcription [13,14,15] have been linked to the disease phenotype; however, the impact on kinase signaling cascades has not been thoroughly investigated. 

Similarly, many other anemias that stem from genetic mutations may have disrupted kinase signaling that has not been closely examined. Basic signaling in red blood cell development has been well characterized for a number of years, but recent revelations using conditional knockout animals and more sophisticated techniques have revealed that the linear cascades, stimulating mostly redundant signaling molecules that are attributed to the cytokines that drive erythropoiesis, do not account for the physiological processes they independently regulate. Nor is it clear how these basic pathways are disrupted or hijacked in disease states. 

The cell cycle is also regulated by a large number of kinases, and cell division is highly regulated during erythropoiesis [16,17,18]. There is evidence that cell cycle progression and differentiation is uncoupled in DBA and is yet another area that deregulated kinases may be impacting erythropoiesis [19]. Metabolism is another kinase-driven pathway disrupted in anemias [20].

In this review, we summarize kinase-signaling cascades initiated during early erythropoiesis, with a particular focus on early erythroid progenitors that are impacted in DBA. Many of these kinase-signaling cascades are well characterized and include Janus kinase/signal transducer and activator of transcription (JAK/STATs), PI3K/Akt, and mitogen-activated protein kinases (MAPKs). In many diseases, it is not the direct deregulation of one of these kinases but rather aberrant deregulation of another kinase that negatively feeds back into the signals initiated by the hematopoietic cytokines [20,21,22,23,24,25,26,27]. While these phosphorylate normally unphosphorylated substrates to trigger pathogenic signals, they frequently disrupt homeostatic pathways. Perhaps the most characterized of these is the activation of the tyrosine kinase cellular Abelson tyrosine kinase (c-Abl) associated with the Philadelphia chromosome fusion event in chronic myeloid leukemia (CML) [28]. Of particular relevance to ribosomopathies, the relatively under-characterized atypical MAPK kinase Nemo-like kinase (NLK) has been shown to be chronically activated in early erythroid progenitors and contributes to the disease phenotype [21,22,23]. Our understanding of how NLK deregulates erythropoiesis is rudimentary but most likely provides only an initial insight into the disrupted kinase network responsible for controlled, healthy red blood cell production.

As with Bcr-Abl, kinases are readily druggable pharmacological targets and are the most successfully targeted class of proteins in medicinal chemistry [6]. Evidence is beginning to emerge that disruption of kinase homeostasis in the erythroid progenitors of DBA patients is contributing to disease pathogenesis [21,22,23]. NLK has overlapping substrate specificity with a number of MAPK members activated in early erythropoiesis and suppression of NLK expression [21,22,23] or activity [21] improves erythroid expansion. Some substrates have been identified in these cells, but understanding the novel pathways initiated, as well as how it disrupts existing pathways, is likely to shed significant insight into our understanding of DBA. As our investigations into kinase signaling in DBA matures, a myriad of other deregulated kinases will likely be identified. Understanding the impacts of these events will undoubtedly improve our understanding of both normal and diseased erythropoiesis.

## 2. Cytokines, Cognate Receptors and Signaling Pathways Regulating Early Erythropoiesis

**Figure 1:**
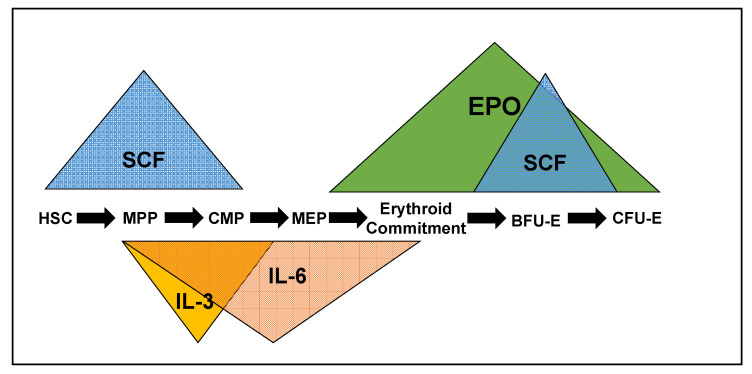
The cytokines Stem cell factor (SCF), Interleukin-3 (IL-3), Interleukin-6 (IL-6), and erythropoietin (EPO) impact erythroid progenitors at different stages of differentiation and can be synergistic, antagonistic or redundant, depending on the progenitor they are stimulating. HSC—hematopoietic stem cell. MPP—multipotent progenitor, CMP—common myeloid progenitor, MEP—megakaryocyte/erythroid progenitor, BFU-E—blast-forming unit-erythroid, CFU-E—colony-forming unit-erythroid.

A number of cytokines, and the kinase signaling cascades they stimulate, drive erythropoiesis, particularly early erythropoiesis. Each of the cytokines impacts progenitors at different stages of differentiation, but there is significant overlap, especially SCF/IL-3/IL-6 and EPO/SCF. The cytokines can also synergize or antagonize one another at different stages of differentiation. Through over-expression or recombinant expression of ligand and receptors in knockout mice, we have determined that each of these cytokines regulates distinct cellular responses, despite significant overlap in the characterized signaling cascades they stimulate (see Figure 1).

**Stem Cell Factor** (SCF) is a dimeric cytokine [6] that binds to the extracellular domain and activates the tyrosine kinase c-kit [3]. Signaling through c-kit is crucial for normal hematopoiesis and a range of other processes, including pigmentation, fertility, gut movement, and aspects of the nervous system [3]. Deregulation of c-kit signaling is linked to cancer and allergy [7]. In hematopoietic cells, c-kit expression is detected in stem and early progenitor populations [8] and lost during differentiation in all lineages except mast and dendritic cells [3]. Activation of c-kit is initiated by dimerization of two c-kit receptors that is brought about by simultaneous binding to the two molecules of the SCF dimer [9]. Dimerization leads to conformational changes that enables the kinase domains of each monomer [6] and trans-phosphorylation of multiple tyrosine residues in both monomers [10]. 

Phosphorylated c-Kit receptors recruit adaptor proteins, including Grb7, Grb10, APS, Lnk, CrkI, CrkII, and CrkL, which, in turn, recruit kinases that are activated [3]. Most notable of these is the p85 subunit of PI3K [10]. Binding of the p85 subunit causes a conformational change that activates the kinase domain of the p110 subunit and PI3K signaling is initiated [12]. Although PI3K phosphorylates phospholipids specifically convert phosphatidylinositol 4,5-bisphosphate (PIP2) to phosphatidylinositol 3,4,5-triphosphate (PIP3) directly, through a series of events, it leads to the activation of Akt that then stimulates a myriad of protein kinase-signaling cascades [12]. 

**Figure 2:**
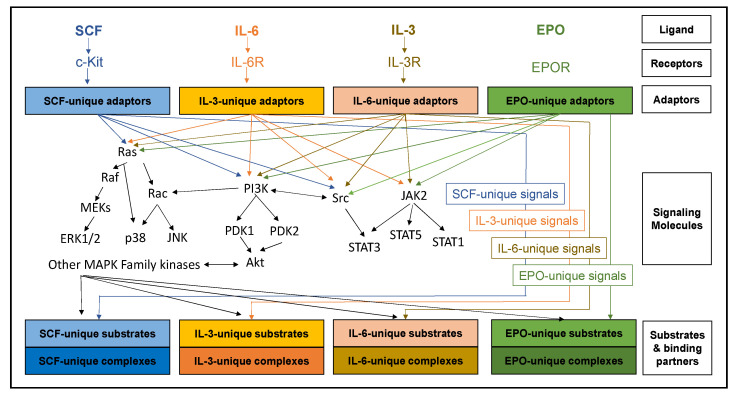
Despite regulating vastly distinct physiological responses to erythropoiesis, SCF, IL- 3, IL-6, and EPO share a large number of classical signaling cascades. The different physiological responses are probably due to nuanced regulation of these cascades with signal specific adaptors, substrates, binding partners, and accessory signaling molecules. The molecular landscape in which a signaling cascade is activated contributes as much to the physiological response as to the signal itself.

The MAPK family of kinases is another large and impactful group of signaling molecules activated upon c-Kit stimulation [3]. ERK1/2 are the most characterized of these, and this pathway is initiated when the small GTPase, RAS, has the guanosine triphosphate (GDP) associated with it exchanged for a guanosine triphosphate (GTP) by a guanine exchange factor [13,14]. A number of the adaptors that associate with phosphorylated c-kit can serve this function (eg, SOS, Vav, Grb2), but the process is tightly regulated and involves a complex of factors [13]. Once bound to GTP, RAS remains active until it hydrolyzes the GTP back to GDP. Active RAS binds the serine/threonine kinase RAF and recruits it to the plasma membrane, where it, in turn, becomes phosphorylated and activated [14]. Activated RAF proteins then amplify the signal by phosphorylating multiple MAPK proteins (including Mek1/2), which are themselves kinases and go on to phosphorylate multiple ERK-like proteins [15]. Many of the substrates are transcription factors in the nucleus [16], but some are powerful signaling molecules, such as ribosomal S6 kinases (RSKs) [16]. There are numerous MAPK family members that signal using a similar template to that of ERK1/2, including the more characterized p38, JNK, and ERK5 signals. All these pathways are activated by SCF, but it is highly probable less characterized members of the family are similarly stimulated [3]. How all these somewhat redundant pathways interact with one another remains under-defined, but it is highly regulated and complex. A summary of SCF signaling is presented in Figure 2.

Phospholipase C is activated downstream of c-Kit [3]. These enzymes hydrolyze the polar head groups of PIP2 to generate diacylglycerol (DAG) and inositol 1,4,5-tris-phosphate (IP3) [29]. These are powerful signaling molecules, but as they are not kinases, they will not be covered here in detail. 

The c-Kit receptor has also been shown to interact with other receptors involved in hematopoiesis, including receptors for EPO, IL-3, IL-7 and granulocyte/macrophage colony stimulating factor (GM-CSF). In some cases, signaling from one cytokine upregulates others [3].

**Interleukin**-**3** (IL-3) is a family of cytokines believed to be important in regulating the growth and development of cells of both the hematopoietic and immune systems. In comparison with other hematopoietic growth factors, IL-3 preferentially supports the proliferation and differentiation of progenitors at early stages of hematopoietic development [30]. IL-3, IL-5, and GM-CSF are bind receptors that are members of the gp140 family. IL-3 acts on the most immature marrow progenitors [31,32,33]. IL-3 is capable of inducing the growth and differentiation of multi-potential hematopoietic stem cells, neutrophils, eosinophils, megakaryocytes, macrophages, lymphoid, and erythroid cells [34]. The activated IL-3 receptor (IL-3R) complex consists of two subunits, a 60–70kDa alpha subunit and a 130–140kDa beta-subunit, bound to a 20–26kDa IL-3 monomer [35]. While no classical tyrosine kinase domains have been identified, evidence suggests tyrosine phosphorylation is at least partially required for signaling [36].

Similar to SCF, IL-3 stimulates PI3K, Src, and MAPK families of kinases [35]. Additionally, IL-3 activates JAK-STAT signals. There are four recognized members of the JAK (Janus kinases) family; JAK-2 appears to be primarily responsible for hematopoietic signaling, although JAK-1 and TYK-2 have been implicated [36,37,38]. JAK proteins associate with the intracellular domain of IL-3R and, upon receptor binding to the IL-3 ligand, JAK proteins are activated. Activated JAKs phosphorylate a number of tyrosine residues on IL-3R that, in turn, serve as docking sites for other signaling molecules, including STATs [33]. Multiple STATs are activated in hematopoiesis, with STAT-1, -3, -5, and -6 most characterized [34]. While JAKs are important for STAT activation, other signaling molecules, such as Src and MAPK family members, are crucial for complete regulatory control [32,38]. 

Although IL-3 stimulates PI3K, RAS/MAPK, and PI3K pathways, the adaptor molecules and activation mechanisms are different. For example, the adaptor Shc binds to the beta-receptor, is rapidly phosphorylated, and becomes associated with Grb2 and SOS. These then exchange GTP to RAS to activate MAPK signaling [39,40,41]. While the highly characterized members of ERK1/2, JNK, and p38 pathways are activated, more detailed analysis will likely reveal regulatory differences between IL-3-mediated and other cytokine-mediated activation of these pathways. Similar to RAS activation, alternative adaptors link IL-3R activation to PI3K stimulation. The adaptor p85 links IL-3R to the p85 subunit of PI3K and downstream signaling such as Akt and p70S6K [34]. A summary of IL-3 signaling is presented in Figure 2. 

**Interleukin-6** (IL-6)—IL-6, IL-11, LIF, and OSM are several of the members of an important family of mediators involved in acute-phase response to injury and infection but are also critical to hematopoiesis [42]. Similar to other cytokines, downstream signaling includes JAK-STATs and MAPK pathways [43]. While there must be significant overlap with other cytokine signals, use of different adaptors and regulators likely provides some novel and crucial input required for differentiation. IL-6 signaling is summarized in Figure 2. 

**Erythropoietin** is first produced in the neural crest cells to stimulate yolk sac erythropoiesis [44,45] but switches to the fetal liver [46] and, primarily, the kidney after birth [47,48]. The mature form of this glycoprotein is 163 amino acids with three potential N-linked glycosylation sites [49]. The erythropoietin receptor, or EPOR, is a single pass transmembrane protein with no recognizable tyrosine kinase domain [50]. Similar to IL-3 and 6, ligand-bound EPOR stimulates JAK-STATs, but also PI3K and MAPKs [50]. Despite EPO signaling through similar pathways as other cytokines, erythroid commitment is heavily reliant upon this cytokine and red blood cell production. The classically defined EPO signaling cascade is summarized in Figure 2. EPO and EPOR null mouse embryos both die early in embryogenesis with a distinct lack of terminal erythroid differentiation, with EPO being critical for promoting the proliferation, survival, and appropriate timing of terminal maturation of primitive erythroid precursors [44]. The receptor itself (EPOR) is upregulated immediately prior to erythroid commitment, and progenitors become responsive to the cytokine [44]. In renal disease, anemia results from the failure of the diseased kidneys to produce adequate amounts of EPO, resulting in subsequent anemia. Administration of recombinant EPO or EPO-stimulating agents increases red blood cell production and relieves anemia these patients [51]. EPO is also used in many anemias even when blood EPO levels are normal to increase stimulation of erythropoiesis including of anemias associated with malignancies, either due to neoplastic bone marrow infiltration or to chemotherapy-related myelosuppression, the anemia of myelodysplastic syndromes and AIDS, the anemia of chronic inflammatory diseases, prematurity, and bone marrow transplantation [51].

In ribosomopathies such as DBA, blood levels of EPO are frequently elevated, yet progenitors are unresponsive to it and no increase in red blood cells occurs [52,53]. This further emphasizes that the disruption in this disease occurs in a very early erythroid progenitor that precedes EPO sensitivity. As with many cytokine signaling pathways that stimulate apparently similar kinase cascades, the particular nuances of the EPO kinase cascade activation are essential for healthy erythropoiesis and is frequently disrupted in disease states, including ribosomopathies. Understanding the specific signaling regulation of this cytokine will likely have significant benefits to human health in the future.

**Kinase roles in erythroid and non-erythroid myeloid differentiation; the Src kinase family.** Once cells are committed to the myeloid lineage, the emphasis on cytokine signaling is reduced, particularly in erythroid progenitors. Non-erythroid myeloid cells require thrombopoietin, GM-CSF, granulocyte colony-stimulating factor (G-CSF), and macrophage colony-stimulating factor (M-CSF) to stimulate progenitors towards appropriate differentiation. In erythroblasts, intracellular kinases contribute to the process [54]. A family of tyrosine kinases critically regulating myeloid lineages is the Src family. The family consists of ten members, and expression can be very cell-type dependent. The expression of a number of members (eg. Lck, Hck, Lyn, Fgr, and Blk) are largely or entirely restricted to hematopoietic cells [55]. The kinases become activated after conformational changes that occur upon binding phosphorylated tyrosine residues on the c-Kit receptor [56]. The activation of specific Src family members is complex, highly regulated, and not completely defined, but relationships with various adaptor and other kinases are critical [3]. The extent, timing, and duration of activation of these kinases is essential for healthy hematopoiesis, as the phosphorylation of downstream substrates interconnect with almost every critical cellular process [56]. 

Another way Src family members can influence different cell types in different ways is by altering subcellular localization. It can be cytosolic or associated with membranes, including plasma, perinuclear, and endosomal membranes, each attributed to different physiological roles [55]. Perhaps the most clinically relevant Src kinase is the Abelson kinase (Abl). In CML, *Abl* is translocated to the *BCR* gene located on chromosome 22 [57]. Inhibition of this deregulated kinase by imatinib mesylate, and subsequent derivatives, has saved many lives and emphasizes the potential of understanding kinase signaling in healthy and diseased myelopoiesis. The potential is not only limited to non-erythroid myeloid progenitors, as Src family kinases are also critical for erythropoiesis. An example is Fyn kinase, which is a modulator of EPO and stress erythropoiesis [58].

## 3. Other Cytokines and Signaling Pathways

The microenvironment in the bone marrow is dynamic, with a complex array of soluble and cell bound ligands that stimulate early erythroid progenitors in a myriad of ways [52]. Our understanding of the niche has increased drastically over recent years, but vast gaps in our understanding of the kinase signaling that is regulating, and being regulated by, these cellular interactions remain. We have discussed the most characterized kinase cascades, but our current understanding of the adaptors, substrates, and binding complexes associated with the activated kinases remains rudimentary. Additionally, it is apparent how critical regulated kinase signaling is to erythropoiesis. Just as apparent is how poorly we understand the mechanistic interactions regulating this network of signals. While it is a challenging task, the potential benefits of comprehensively understanding how these kinases interact are significant. As we move forward, we must not think of signaling molecules as on or off, or as belonging to discreet pathways, but rather interconnected pathways enhanced or suppressed to varying degrees by a multitude of signals. Perhaps just as intriguing is the concept that different signals switch the proteins associated with active kinases that result in modified cell localization of the activated kinases. Kinases and kinase signaling are nuanced and not binary.

## 4. Kinases and Anemia

Despite being highly influenced by kinases, relatively few erythropoiesis deficiencies are currently attributed to deregulated kinase activity. The most characterized of these are pyruvate kinase, DYRK3, mTORC1, p38/JNK, and NLK [21,22,23,25,59,60,61].

**Pyruvate kinase**—Central to red blood cell production is glycolysis, and the conversion of phosphenolpyruvate to pyruvate yields 50% of the ATP required for erythropoiesis [61]. The enzyme that catalyzes this reaction in pyruvate kinase. Mutations in this gene give rise to pyruvate kinase deficiency and are characterized by hemolysis and non-spherocytic anemia [20,24]. Although not strictly a signaling kinase, this emphasizes the broad role kinases play in erythropoiesis.

**Dual-specificity tyrosine-regulated kinase-3** (DYRK3)—The expression of DYRK3 is limited to erythroid progenitor cells and the testis. Studies in mice suggest that the activation of this kinase during induced anemia contributed to reduced erythropoiesis. One possible mechanism of action is that DYRK3 inhibits the NFAT (nuclear factor of activated T cells) transcriptional response pathway to modulate the essential erythroid transcription factor Klf1 [25].

**Mammalian target of rapamycin** (mTOR)—Ribosomopathies, and the reduced ribosome function associated with them, clearly indicate the importance of protein translation in erythropoiesis. A critical regulator of this process is mTOR [62]. DBA models [63] and patients [64] can be significantly improved upon treatment with the stimulator of mTOR, leucine, further implicating the importance of this master kinase in regulating erythroid development. Inhibitors of mTOR have also been demonstrated to negatively impact erythropoiesis [64], although inhibitors could also improve anemia in some conditions [26,65]. As this kinase is regulated by a complex array of factors, it may contribute in multiple ways under different conditions. The regulatory subunit of mTOR, RAPTOR, is phosphorylated by activated NLK in DBA models [21], suggesting that NLK may be contributing to mTOR deregulation in erythroid progenitors. 

**Heme-regulated elF2α kinase**—The kinase is also known as the heme-regulated inhibitor (HRI) and is activated in the heme deficiency that occurs in microcytic hypochromic anemia [66]. The activated kinase phosphorylates elF2α to inhibit translation of certain mRNAs (especially globin) and enhance translation of other mRNAs (such as ATF4). This pathway also represses mTORC1 and impacts mitochondrial function [66].

**JAK2–JAK2V617F**—This is a point mutation of the JAK2 gene and results in myeloproliferative disorders with a polycythemia-like phenotype and increased erythropoietin-independent red blood cell production and splenomegaly [67]. While most aberrantly activated kinases that contribute to disrupted erythropoiesis in human health do not belong to the signaling pathways of the classical hematopoietic cytokines, this mutation of the JAK2 kinase is a rare example of the direct disruption of such kinases in human disease. This mutation contributes to 100% of cases of polycythemia vera along with many patients with essential thrombocythemia and primary myelofibrosis [67].

**ERK/SAPK/JNK/p38**—Within the MAPK family of kinase are subfamilies, including ERK1/2, ERK5, p38, JNK, and SAPK. Additional atypical and less conserved members are also present [68]. Of these ERK1/2, p38α and JNK1 are the most characterized, with most members being poorly characterized. In hematopoiesis ERK1/2, p38α and JNK1 are activated in response to erythropoiesis cytokines [3,34,48], but it is highly likely that regulation of other MAPK family members occurs but has not yet been characterized. However, it has been revealed that p38α and JNK1 restrains erythropoiesis [69], and p38 is required for the production of erythropoietin by bone marrow cells [4]. Deregulation of the p38 pathway is partially responsible for apoptosis in Fanconi anemia [27]. Early work suggests erythroid proliferation is dependent on ERK1/2 signaling, while differentiation is mediated primarily through p38/JNK signaling [70]. Deregulated signaling of the SAPK family of kinase is linked to hematopoiesis in Fanconi Anemia [71]. Although correctly regulated MAPK signaling is evidently essential to erythropoiesis, our current limitations in understanding of the complexities of these signaling networks in healthy and disease states of erythropoiesis impairs our ability to utilize them as therapeutic targets. A better understanding of the full complement of MAPK family members should rectify this. 

**Nemo-Like Kinase** (NLK)—The atypical MAPK kinase NLK is chronically hyper-activated in DBA and contributes to disease pathogenesis [21,22,23]. NLK expression is moderate in HSCs and is downregulated in non-erythroid progenitors by the upregulation of miR-181 [21,72]. While this miRNA critically regulates the differentiation of MEPs into megakaryocytes [73], it also leads to degradation of NLK mRNA [21,22,23,72]. NLK null mice die late in pregnancy or just after birth due to compromised lung development. Depending on the mouse strain, the hematopoietic system ranges from unaffected to severely compromised [74]. The bone marrow niche is also disrupted with reduced fat tissue and stromal cells. NLK regulates the Wnt pathway but can also influence STAT3 and interferon signaling [74]. 

While NLK expression appears dispensable for normal erythroid development, in DBA NLK becomes activated [21,22,23]. The upstream regulators are, as yet, uncharacterized, but are dependent on the increased p53 expression associated with the disease [21]. As a MAPK member, NLK shares many substrates with other MAP kinases, albeit with differing kinetics [69]. The exact substrates responsible for erythroid defects are not fully characterized, but the degradation that accompanies c-Myb phosphorylation and inhibition of the translation regulator mTORC1 are likely effectors [21]. The activation of NLK occurs in all DBA genetic mutations examined to date (although this is not exhaustive) and may therefore constitute a common therapeutic target in DBA patients, irrespective of the genetic mutation carried. Unfortunately, NLK suppression does not completely rescue erythroid expansion but does improve erythropoiesis by 2–6-fold, depending on the in vitro model tested [21,22,23]. As p53 is upregulated in a number of anemias, NLK or other MAPK family kinases may be similarly activated in these disorders.

This list is far from comprehensive, and advancements in our understanding of the regulatory mechanisms of erythropoiesis will certainly reveal a growing list of deregulated kinases that may serve as therapeutic targets to reduce the burden of anemic diseases. 

## 5. Summary

We have learned a tremendous amount about the major signaling molecules and cascades initiated by classical hematopoietic cytokines such as SCF, Il-3, IL-6, and EPO. However, there is a significant overlap, in which kinase cascades are initiated by each, despite each having very distinct and dramatic physiological impacts on erythropoiesis. To understand how these cytokines impact erythropoiesis, we need to delve deeper and better understand the complexities of kinase signaling in erythroid progenitors. As so many other cellular processes that are critical to erythroid expansion are also regulated by kinases we also need to understand the interplay between these kinase and how deregulation in one impacts the entire network. Only once we have a better understanding of kinase signaling in normal erythropoiesis will we be able to translate this understanding for clinical benefit in ribsomopathies such as DBA and other anemias. 

Kinases offer hope as therapeutic targets in a wide range of human diseases. As erythropoiesis is so dependent on kinase signaling cascades, understanding how these cascades are influenced in the disease state will, almost without doubt, reveal therapeutic strategies to improve patient outcomes. In particular, kinases that are deregulated in multiple genetic blood disorders, for example NLK being activated in ribosomopathies [21,22,23], offer particular value as common targets to multiple genetic disorders. The challenge will be understanding the deep, interconnected network that has developed from many years co-evolving into the highly integrated and regulated system and how the manipulation of one kinase will impact the network as a whole. The potential reward of understanding such complexities could greatly benefit human health. 

## Data Availability

No new data were created or analyzed in this study. Data sharing is not applicable to this article.
